# Efficacy of miltefosine compared with glucantime for the treatment of cutaneous leishmaniasis: a systematic review and meta-analysis

**DOI:** 10.4178/epih.e2019011

**Published:** 2019-03-31

**Authors:** Sohrab Iranpour, Ali Hosseinzadeh, Abbas Alipour

**Affiliations:** 1 Student Research Committee, Department of Epidemiology, Faculty of Public Health and Safety, Shahid Beheshti University of Medical Sciences, Tehran, Iran; 2 Department of Community Medicine, Faculty of Medicine, Ardabil University of Medical Sciences, Ardabil, Iran; 3 Research Center for Modeling in Health, Institute for Future Studies in Health, Kerman University of Medical Sciences, Kerman, Iran; 4 Department of Community Medicine, Faculty of Medicine, Mazandaran University of Medical Sciences, Mazandaran, Iran

**Keywords:** Efficacy, Miltefosine, Glucantime, Cutaneous leishmaniasis, Systematic review, Meta-analyses

## Abstract

Cutaneous leishmaniasis (CL) is most common form of leishmaniasis and is characterized by ulcerative skin lesions. The objective of this study was to conduct a systematic review and meta-analysis of clinical trials that compared the efficacy of miltefosine and glucantime for the treatment of CL. We searched the following databases: Cochrane, PubMed, Embase, Scopus, Web of Science, ProQuest, Cochrane Central Register of Controlled Trials, International Clinical Trials Registry Platform search portal of World Health Organization, Sid, Irandoc, Magiran, and clinicaltrials.gov. We used keywords including “miltefosine,” “glucantime,” and “*Leishmania*.” The quality of studies was assessed using the Cochrane risk of bias tool. A random-effects model was employed for the analysis. We assessed heterogeneity by the chi-square test and the I^2^ index statistic. When heterogeneity was present, meta-regression analyses were performed. The Egger method was used to assess publication bias; when it was significant, the trim-and-fill method was used to test and adjust for publication bias. A total of 1,570 reports were identified, of which 10 studies were included in the meta-analysis. In the meta-analysis, there was no significant difference between the efficacy of miltefosine and glucantime; however, subgroup analysis showed that, regarding parasite species other than *Leishmania braziliensis*, miltefosine was significantly superior to glucantime (intention to treat; relative risk, 1.15; 95% confidence interval, 1.01 to 1.32). In the meta-regression, only the glucantime injection type was significant at the p=0.1 level. The Egger test found statistically significant publication bias; however, including the 3 missing studies in the trim-and-fill analysis did not change the results. This meta-analysis found that miltefosine seems to be more effective than glucantime, at least in species other than *L. braziliensis*, for treating CL.

## INTRODUCTION

Leishmaniasis is a protozoan parasitic infection that, depending on the infecting species of *Leishmania*, can manifest in different forms, including cutaneous, mucocutaneous, or visceral leishmaniasis [1]. Infections occur all over the world; however, it is endemic in 98 countries, predominantly tropical and subtropical. Over 350 million people are at risk globally, with 1.3 million new cases each year and 20,000 to 40,000 deaths annually [2,3]. Cutaneous leishmaniasis (CL) can be caused by a variety of species, including *Leishmania braziliensis, Leishmania amazonensis, Leishmania aethiopica, Leishmania mexicana, Leishmania guyanensis, Leishmania panamensis, Leishmania peruviana, Leishmania tropica and Leishmania major*. CL, characterized by ulcerative skin lesions, is the least fatal but most common form of the disease [1]. CL is widely distributed; however, 70% to 75% of the estimated global incidence is accounted for by 10 countries, including Brazil, Colombia, North Sudan, Iran, Afghanistan, Algeria, Syria, Ethiopia, Costa Rica, and Peru [2]. In such countries, although of the local population seem to be developing some immunity [4], others such as travelers, tourists, workers, and military troops are more at risk and may also inadvertently carry the disease to non-endemic areas [5,6]. Although CL is a self-limited disease, in some cases it may be severe and lead to mucosal disease or disseminated leishmaniasis [7-9]. Since the 1940s, pentavalent antimonials (meglumine antimoniate [MA] and sodium stibogluconate) have been considered to be the first line of treatment for leishmaniasis [10]; indeed, they are widely used for the treatment of all forms of leishmaniasis [11]. Some of the well-known problems associated with using pentavalent antimonials include the frequency and severity of adverse symptoms and difficulties in their administration [11]. With regard to the former factors, these drugs are very toxic and may affect organs such as the pancreas, liver, kidney, and the hematological system [12]. Some serious side effects include cardiac arrhythmia, pancreatitis, myalgia, arthralgia, liver enzyme elevation, the possible need for repeated parenteral injections [13-16], fever, and headache [17]. Therefore, it is clear that the use of pentavalent antimonials could pose life-threatening risks. Additionally, drug resistance has become widespread [13]; in previous studies, a decrease in the efficacy of MA and a decrease in sensitivity of *Leishmania* parasites to antimonials have been reported [10,18-21]. An increasing number of Iranian patients are unresponsive to MA as a first-line treatment for leishmaniasis; indeed, it has been demonstrated that in about 40% of CL cases, there is no desirable beneficial response to MA during the first course of treatment [22,23]. Furthermore, in certain regions where antimony compounds have been used for a long time, 40% of cases show disadvantages in terms of drug toxicity and subsequent clinical resistance [15,24,25]. The decreasing sensitivity of *Leishmania* parasites to MA and the potential irregular adherence to the daily schedule of the parenteral route during the first 20 days are the main factors underlying the decreasingly desirable response rate to MA [26,27]. Therefore, to better control CL, it is necessary to develop new therapeutic strategies with a higher efficacy and safety rate, coupled with better patient adherence [28]. This would require a greater push towards more productive research into therapeutic alternatives for the treatment of leishmaniasis [17]. Miltefosine, a phosphatidylcholine analogue, is an antileishmanial oral drug that has been shown to be >95% curative for visceral leishmaniasis in India [29]. A high cure rate of CL (91%) has also been demonstrated after oral treatment with miltefosine [30,31]. In one study, miltefosine cured 88% of patients with aggressive *L. major* infections that did not respond to intralesional antimony [32]. Miltefosine is a safe and effective oral treatment for CL [33-35] that could be used as an option for CL therapy instead of MA. However, findings from studies that have compared MA and miltefosine are inconsistent. For instance, in some studies, the therapeutic efficacy of oral miltefosine was not significantly different from that of MA [11,33,36-38]. In other studies, the efficacy of miltefosine was statistically significantly superior to that of MA in the treatment of CL [28,35,39]. However, another randomized clinical trial (RCT) found a significant difference favoring MA [12]. In a systematic review and meta-analysis published in 2013 that included 5 studies conducted in American countries, no significant difference was found between miltefosine and MA in the complete cure rate at 6 months. However, when pooling 2 studies focused on *L. panamensis* and *L. guyanensis* species, a significant difference favoring miltefosine was found in the rate of complete cure at 6 months [40]. The objective of this paper is to present a systematic review and meta-analysis of clinical trials that compared the efficacy of miltefosine and MA in the treatment of CL in countries throughout the world.

## MATERIALS AND METHODS

In this study, we adhered to the guidelines of the 2009 PRISMA (Preferred Reporting Items for System reviews and Meta-Analyses) statement for reporting systematic reviews and meta-analyses of RCTs.

### Literature search and data sources

Literature searches of electronic databases from 1991 to July 31, 2017, were conducted in August 2017. We searched the Cochrane Library, PubMed, Embase, Scopus, Web of Science, ProQuest, Cochrane Central Register of Controlled Trials, International Clinical Trials Registry Platform search portal of WHO, 3 Persian-language databases (Sid, Irandoc, and Magiran), and clinicaltrials.gov. In addition, hand-searching of the references of the included articles and previous reviews and meta-analyses constituted secondary search strategy to find other eligible trials. Gray literature papers, book chapters, and the main journals in the field of leishmaniasis were also searched. There were no language restrictions in the searches. All retrieved references were managed with a reference manager program (EndNote).

This search used the following keywords: “miltefosine,” “glucantime,” and “Leishmania”.

The PubMed systematic search strategy is presented in Supplementary Material 1. This search strategy was amended with adjustments in vocabulary and syntax for each database.

### Interventions and comparisons

The interventions were miltefosine and MA through any route of administration. The primary efficacy outcome of interest was the complete cure rate at 6 months.

### Systematic review process

For screening, titles and abstracts from the primary search were independently assessed by 2 reviewers (SI and AH) for the full text of the studies according to the selection criteria. For further screening, the full text of all potentially eligible studies was then assessed using the inclusion and exclusion criteria by the same 2 reviewers. Disagreements were solved by discussion and consensus.

The original search included all study designs, but only clinical trials were included in the analyses for this paper. Studies were included if they involved patients of any age of male and/or female sex, reported efficacy in human participants, and contained enough information to extract data for an intention-to-treat (ITT) analysis. Articles were excluded if they described animal, *in vitro*, or *in vivo* experimental studies or if they were expert opinion papers.

### Data extraction and risk of bias assessment

The relevant data from the studies were extracted independently by 2 authors (SI and AH) using a pre-designed data extraction form and were checked for accuracy and completeness by a third reviewer (AA).

Two authors subsequently met to discuss their findings to resolve any discrepancies among the extracted data; any persistent discrepancies were resolved by consultation with a third author (AA).

The extracted data included publication characteristics (e.g., name of author, year, study design, and country), inclusion and exclusion criteria, sample size, population characteristics (e.g., patients’ age and sex), intervention details (dose of drugs, route of administration, and period of treatment), outcomes, and design (length of follow-up, randomization, blinding, and allocation concealment). To obtain an ITT estimate, conservatively assuming that subjects lost to follow-up did not change their baseline consumption levels, the total number of patients in each group in baseline and the number of patients who met the response criteria were recorded.

The quality of the included studies was assessed by 2 authors (SI and HA) independently. Discrepancies were resolved in consultation with the third reviewer (AA). We used the Cochrane risk of bias tool to assess the quality of studies. This tool includes 7 specific domains: random sequence generation, allocation concealment, blinding of participants and personnel, blinding of outcome assessment, incomplete outcome data, selective reporting, and other sources of bias [41]. Studies were classified as having a low, high, or unclear risk of bias. Any discrepancies between reviewers were resolved in consultation with the third author (AA) until a consensus was reached.

### Data analysis

Efficacy analyses were based on the reported numbers of confirmed cases of CL in each study and were performed for outcome measures if at least 2 studies could be pooled together.

We presented summary estimates from efficacy analyses primarily as relative risk (RR) to assess the strength of the effect; however, as an indicator of clinical significance, we also reported the risk difference (RD). All are reported with 95% confidence intervals (CIs). The meta-analyses were performed using a fixed-effects or random-effects method with inverse-variance weights and the DerSimonian-Laird estimator, respectively.

We assessed and quantified heterogeneity among studies using the chi-square test, with significance set at p-value<0.05, and the I^2^ index, which is the percentage of the variation in the effect size estimation attributed to heterogeneity [42].

If heterogeneity was found to be present, meta-regression analyses were performed to identify possible relationships between efficacy and factors such as the sex ratio, sample size, study quality, and country location (New World or Old World).

A sensitivity analysis was used to verify the reliability of the results. A post hoc influence or sensitivity analysis was conducted by leaving out 1 study from the meta-analysis at a time and checking the consistency of the combined effect estimate.

Publication bias was assessed through visual inspection of the asymmetry of funnel plots. The Egger method, which is based on the asymmetry of funnel plots, was used to assess the influence of publication bias and, when significant evidence of publication bias was found, the trim-and-fill method was used to test and adjust for publication bias. The meta-analyses were performed using Stata version 12.0 (StataCorp., College Station, TX, USA).

## RESULTS

**Figure 1.**
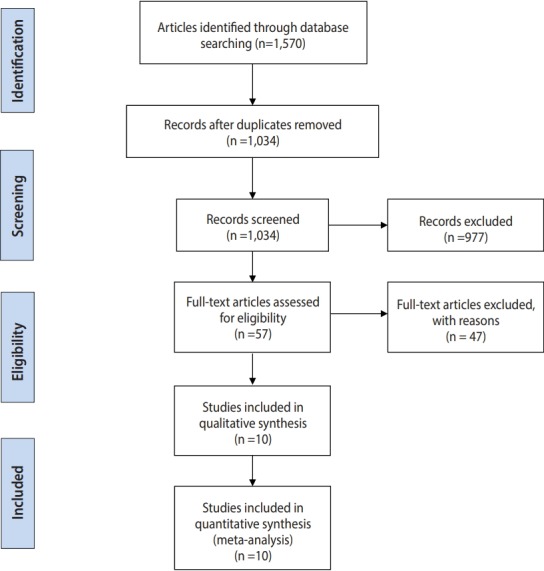
PRISMA (Preferred Reporting Items for System reviews and Meta-Analyses) flow diagram systematic search and review process.

The flow diagram presented in Figure 1 summarizes the study selection process.

A total of 1,570 reports were identified from the databases and by manual searching. After duplicates were removed, 1,034 papers remained, and after screening titles and abstracts, 57 studies were selected as potentially eligible to be included. After referring to the full texts, we ultimately included 10 studies (involving 1,006 participants; 550 in the miltefosine group and 456 in the MA group) in the efficacy meta-analyses. The characteristics of the included studies are presented in Table 1.

The studies were published between the years 2007 and 2014 (median, 2010). The mixture of geographic locations of studies was not very broad; 3 studies were conducted in Iran, 2 in Colombia, 2 in Brazil, 2 in Bolivia, and 1 in Pakistan. Except for 1 study [11] that was non-randomized, all studies were randomized parallel-group trials, and majority of the studies were not blinded. Most studies used an individual randomization design, but 3 used block randomization [12,33,36]. Seven studies had a mix of males and females [11,28,33,35,36,38,39], 1 study had only males [12], and in 2 studies the sex ratio was unclear [34,37]. The sample size ranged from a minimum of 30 to a maximum of 288. Most studies recruited patients aged >12, while 1 RCT-enrolled only children (age <12) [36]. Regarding parasite species, the majority of studies included *L. braziliensis*. One study provided a topical ointment intervention [39], whereas all the other studies provided oral miltefosine and injected MA. Oral miltefosine was compared in 7 studies with intramuscular injections of MA [11,12,33,34,36-38] and in 2 studies with intravenous MA [28,35]. The follow-up of the included studies ranged from 1 month to 6 months.

The risk-of-bias evaluations of the included studies are presented in Table 2. Most of the included studies were judged to have an unclear risk of selection bias, since the reporting of methods for randomization and allocation concealment was limited. Regarding the risk of performance bias due to non-blinding of patients, personnel, or outcome assessors, most of the included studies were judged at high risk for selection bias. Most studies were judged to be at low risk for attrition bias. Overall, studies were classified as having a high, low, or unclear risk of bias according to all components.

In 1 study that did not report frequencies for the outcome of interest, the frequency was calculated based on the percentages reported in the article [34].

The included studies reported results for different follow-up periods.

Except for the 6-month post-treatment follow-up, for which both fixed-effects and random-effects methods were used for the meta-analyses, we used the random-effects method for all other post-treatment follow-up periods.

The results of the analyses of different follow-up periods are presented in Table 3.

**Figure 2.**
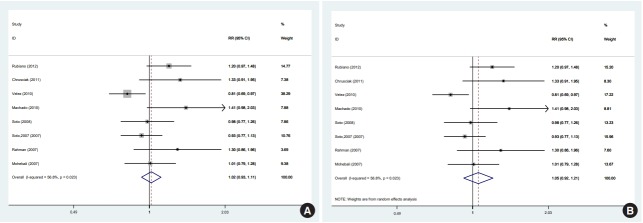
Meta-analysis of the eight studies evaluating miltefosine compared to meglumine antimoniate in the rate of complete cure at 6 months of follow up (A) fixed-effects model, (B) random-effects model. RR, relative risk; CI, confidence interval.

**Figure 3.**
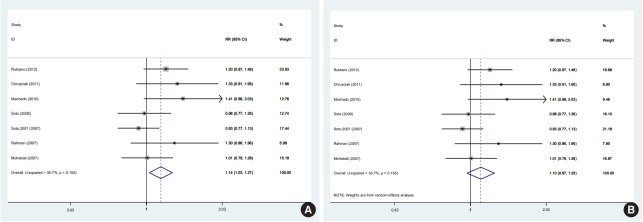
Meta-analysis of the seven studies evaluating miltefosine compared to meglumine antimoniate in the rate of complete cure at 6 months of follow up (A) fixed-effects model, (B) random-effects model. RR, relative risk; CI, confidence interval.

In 1 of the 6 studies reporting the end-of-treatment cure rate and the 4 studies reporting the results at a 1-month post-treatment follow-up, the intervention was an ointment [39]; when excluding this study, the result did not change at either time point. In a meta-analysis of the 5 studies [11,12,33,36,38] that reported results at a 3-month post-treatment follow-up, there was no significant difference between the efficacy of miltefosine and that of MA. One of the 5 studies was conducted in a special population (the Colombian Army) different in some characteristics, such as age range, sex (only male), and activity. In a sensitivity analysis excluding that study, which received the highest weight [12], miltefosine was significantly superior to MA in the complete cure rate at 3 months. There were 8 studies [11,12,28,33-36,38] that reported results from a 6-month post-therapy follow-up, which was the main focus of the literature and of this study. In a meta-analysis of these 8 studies [11,12,28,33-36,38] with a fixed-effects and random-effects models, there was no significant difference between the efficacy of miltefosine and that of MA (Figure 2). However, in a sensitivity analysis excluding the study [12] with the highest weight (37.5%), miltefosine was significantly superior to MA (Figure 3A).

Furthermore, using a random-effects model in the sensitivity analysis and excluding the study [12] with the highest weight (17.2%), miltefosine was as effective as MA in the complete cure rate at 6 months (Figure 3B).

The results of analyses regarding parasite species are presented in Table 4.

There was no significant difference between the efficacy of miltefosine and that of MA with a random-effects model according to parasite species when the studies with a 6-month follow-up period were pooled.

There was no significant difference among any of the 4 studies with *L. tropica/major, L. panamensis, L. major, and L. guyanensis* species. Likewise, when the 4 studies with *L. braziliensis* were pooled [12,28,34,38], there was no significant difference between the efficacy of miltefosine and that of MA. However, in a subgroup analysis excluding the studies with *L. braziliensis*, with the other 4 studies pooled using a fixed-effects model, miltefosine was significantly superior to MA (ITT: RR, 1.18; 95% CI, 1.02 to 1.37; RD, 0.12; 95% CI, 0.02 to 0.22: 293 participants) [11,33,35,36], and the superiority of miltefosine persisted when a random-effects model was used (ITT: RR,1.15; 95% CI, 1.01 to 1.32; RD, 0.11; 95% CI, 0.01 to 0.21: 293 participants).

Because the I^2^ statistic was found to be 56.8% (chi-square=16.20; df=7; p=0.023) in our review, a meta-regression analysis was conducted to investigate potential sources of heterogeneity, including the sex ratio, sample size, study quality, country type (New World or Old World), and MA injection type. Table 5 shows the results of the meta-regression between log(RR) and potential variables. As shown in Table 5, the results of the meta-regression were not statistically significant, except for MA injection type, at the p=0.1 level.

Publication bias was assessed using funnel plots (Supplementary Material 2), which found asymmetry. The Egger test (t=3.35; p=0.015) found statistically significant publication bias (Supplementary Material 3). The trim-and-fill analysis revealed that 3 studies had been missed or trimmed (Supplementary Material 4). The trim-and-fill analysis demonstrated that including the missing studies did not change the results, and there was no significant difference between groups in either a fixed-effects model (ITT: RR, 0.96; 95% CI, 0.88 to 1.04: 11 studies) or a random-effects model (ITT: RR, 0.97; 95% CI, 0.82 to 1.12: 11 studies).

## DISCUSSION

In this study, a PRISMA-compliant systematic review and meta-analysis was conducted based on clinical trials comparing miltefosine and MA in order to evaluate their efficacy in treating CL. In this meta-analysis, 10 clinical trials with 1,006 participants were included, exclusively comprising studies comparing miltefosine and MA as single-drug interventions. We observed significant variation in follow-up times. The 8 studies with 6-month follow-ups were the main focus of this study [11,12,28,33-36,38]. Using a random-effects model on an ITT basis, in the meta-analysis of 8 studies comparing miltefosine with MA for CL, we found no significant difference in the cure rate at 6 months. Using a random-effects model, the meta-analysis yielded an overall RR of 1.05 (95% CI, 0.91 to 1.20) and an RD of 0.04 (95% CI, -0.05 to 0.15; I^2^=60%). Visual inspection of the forest plot indicated significant heterogeneity between the study with the largest sample size [12] and the remaining studies. The study with the largest sample size was conducted on adult males serving in the Colombian Army, and it was the primary contributor to the high heterogeneity observed among the included studies; when this study was removed, the heterogeneity among the different studies decreased significantly. Differences in the sample size, age range, sex ratio, and other factors between that study and other studies might have contributed to the high heterogeneity. However, the main effect remained the same in the random-effects model, but changed in the fixed-effects model to favor miltefosine.

Some evidence suggested that the species of parasite is a factor influencing the cure rate of drugs and that the differences in the therapeutic response between the studies carried out in different regions may be partially attributed to the *Leishmania* species [26,30]. However, no clear consensus emerged regarding the *Leishmania* species.

Interestingly, in our study, in all the included studies with species other than *L. braziliensis*, miltefosine was superior, with no significant difference in the cure rate at 6 months. Although in subgroup analyses based on parasite species, no significant differences were detected in any of the subgroups in the cure rate at 6 months, when pooling the 4 studies with species other than *L. braziliensis* (including *L. panamensis, L. guyanensis, L. tropica*, and *L. major*) to increase the power, there was a significant difference favoring miltefosine in the cure rate at 6 months with no heterogeneity. When pooling studies with *L. braziliensis* [12,28,34,38], MA was superior with no significant difference in the cure rate at 6 months with zero heterogeneity. Therefore, the subgroup analyses conducted to evaluate species-specific efficacy suggest that miltefosine may be superior to MA in species other than *L. braziliensis* and non-inferior in *L. braziliensis*. This demonstrates that the effect of drugs likely depends on the parasite species.

We found similar results for the 4 studies with a 3-month follow up period. After excluding 1 study that included *L. braziliensis*, and pooling the remaining 3 studies with other species, miltefosine was significantly superior to MA.

These findings are similar, to some extent, to the results of a study conducted in Brazil comparing the therapeutic response of CL due to *L. braziliensis* and *L. guyanensis* to MA, which showed that *Leishmania* species was an important factor in predicting the outcome of CL treated with MA; in particular, the failure rate was higher in patients infected with *L. guyanensiss* [26].

The findings of the present study are to some extent inconsistent with previous systematic reviews and meta-analyses [40]. The conflicting results between our meta-analyses and previous meta-analyses may be due to the inclusion of updated studies from the Old World that were not included in previous meta-analyses. It is important to bear in mind that the majority of the studies in previous meta-analyses included *L. braziliensis*, whereas the updated studies in our review mainly analyzed other species. Nonetheless, in subgroup analyses we found that miltefosine was non-inferior to MA in *L. braziliensis*, similarly to previous meta-analyses, and superior in species other than *L. braziliensis*.

Meta-regressions were conducted for the sex ratio, sample size, study quality, and country type (New World or Old World). Among these characteristics, only the route of administration of MA was significant. Performing meta-regressions adjusted for confounding variables was limited by the relatively small number of studies.

### Publication bias

The analysis of publication bias was limited by the relatively small number of studies. With this limitation in mind, publication bias was assessed through a funnel plot, the Egger test, and a trim-and-fill analysis. The funnel plot and Egger test showed the presence of publication bias in the included studies. However, the trim-and-fill analysis demonstrated that the impact of publication bias was within an acceptable range, and the inclusion of the missing studies did not change the overall conclusions.

Overall, the findings of our study show that although the cure rate of miltefosine treatment compared with MA varied among *Leishmania* species, miltefosine was at least as good as MA in treating CL caused by *L. braziliensis* and superior in treating leishmaniasis caused by other species. In terms of the most important advantage of miltefosine (its comfort for patients) [33], the practical importance of this finding is that miltefosine can be administered for CL as an oral agent that will be approximately as effective as standard injections of MA.

Some mild adverse gastrointestinal effects such as anorexia, nausea, abdominal pain, diarrhea, and vomiting are common and were reported with miltefosine [29-31]. However, they do not usually require suspension of treatment [12]. Therefore, miltefosine could be a safe and effective alternative to MA and could be especially helpful in regions where parasites are resistant to MA.

One major problem with miltefosine is teratogenicity in reproductive-age female. Using contraception during therapy and for 4-5 months after treatment completion is necessary due to the long half-life of miltefosine [43].

The major limitation of our study was its relatively small sample size and the paucity of relevant studies, which restricted the assessment of heterogeneity and publication bias. Neither allocation concealment nor blinding was performed in the majority of the included studies.

## CONCLUSION

This meta-analysis found that miltefosine seems to be more effective than MA in treating CL, at least in species other than *L. braziliensis*.

## Figures and Tables

**Table 1.** The characteristics of included studies

First author and year of publication [Ref]	Methods	Participants and criterion	Age range or mean (yr)	Interventions	Parasite species	Outcomes and fallow-up	No. of cases	
							Miltefosine	MA
Vélez, 2010 [12]	Open label, RCT (phase III) in five military health establishments located in Colombia	- Adult males serving in the Colombian Army	19-38	MA: intramuscularly at a dose of 20 mg/kg BW/d for 20 d	*L. panamensis, L. braziliensis*	Cure rate 3 and 6 mo after; failure; recurrence; reinfection rescue therapy	145	143
		- Inclusion criteria: confirmed parasitological diagnosis of leishmaniasis; received no treatment of the current infection during the past 6 wk; normal renal, hepatic, pancreatic, and hematological functions		Miltefosine: 50 mg orally 3 times/d for 28 d				
		- Exclusion criteria: serious concomitant illnesses; lesions with mucosal involvement; disseminated CL (presence of 10 or more cutaneous lesions and a negative Montenegro skin test).						
Rubiano, 2012 [36]	Multicenter, open label, RCT conducted in 3 geographic locations in Colombia	- Males and females	2-12	MA (81 mg Sb/mL) at 20 mg Sb/kg/d intramuscular for 20 consecutive days	*L. panamensis, L. guyanensis, L. braziliensis *(n=1)	Cure rate therapeutic failure during 3 and 6 mo after, parasitologic response; adverse events.	58	58
		- Inclusion criteria: children aged 2-12 yr with parasitologi- cally confirmed CL		Miltefosin orally (10 mg miltefosine/capsule) at 1.5–2.5 mg/kg/d by mouth during 28 consecutive days, divided into 2 or 3 daily doses				
		- Exclusion criteria: weight >10 kg, mucocutaneous disease, use of anti-*Leishmania* medications during the month prior to diagnosis, medical history of cardiac, renal, or hepatic disease, menarche, and and baseline values for hemoglobin, amylase, aspartate AST, ALT, creatinine, and serum urea nitrogen outside the normal range						
Mohebali, 2007 [33]	Open label, RCT conducted in Golestan province, in the northeast of Iran	- Patients with a clinical diagnosis of CL (males and females)	Miltefosine: 20.2	MA intramuscularly at 20 mg SbV 5/kg BW daily for 14 d	*L. major*	Cure rate failure relapse 2 wk 3 and 6 mo after; adverse event	32	31
		- Inclusion criteria: observation of Leishman bodies (amastigotes) in dermal lesions, no previous use of anti-leishmanial drugs; no previously confirmed leishmaniasis (by scar or clinically compatible history)	MA: 16.8	Miltefosine orally at a dosage of ~2.5 mg/kg daily for 28 d				
		- Exclusion criteria: pregnancy or lactation, acute or chronic medical condition and history of allergy; female patients of childbearing age were included after giving consent for effective contraception during therapy and until 3 mo thereafter						
Rahman, 2007 [11]	Non-randomized, open label trial at military hospital, Rawalpindi, in Pakistan	- Civilian and soldiers who acquired infections mostly in the endemic areas (males and females)	≥12	MA intramuscularly in a dose of 20 mg/kg/d for 28 d; orally miltefosine in a dose as close to 2.5 mg/kg/d for 28 d	*L. tropica/major*	Cure rate, at 3 and 6 mo	15	15
		- Inclusion criteria: aged 12 yr or older, parasitologically confirmed CL and without any other significant concomitant disease, the duration of the lesions ranged from 2 wk to 3 mo, normal blood counts, liver enzymes and renal function tests before the onset of therapy						
		- Exclusion criteria: children less than 12 yr of age, females of childbearing age and pregnancy or lactation						
Machado, 2010 [28]	Open label, randomized trial at the health post of Corte de Pedra, located 260 km southeast of Salvador, the capital of Bahia, Brazil	- Males and females	4 -65	MA intravenously at a dose of 20 mg SbV/kg/d for 20 consecutive days (maximum daily dose of 3 ampoules or 1,215 mg/Sbv)	*L. braziliensis*	Cure rate, at 2 wk, 1, 2, 4 and 6 mo; relapses; adverse events	60	30
		- Inclusion criteria: presence of a typical ulcerated lesion and a positive Montenegro intradermal skin test in a subject living in the endemic area; age 2-65 yr; a maximum of 5 ulcers with no more than 2 body regions involved; lesion size between 10 mm and 50 mm in a single dimension; a period of less than 90 d from the onset of the first ulcer; all subjects were submitted to a Punch biopsy to obtain material for *Leishmania* culture and PCR		Miltefosine orally at the total target daily dosage of 2.5 mg/kg of BW (maximum daily dose of 150 mg) for 28 consecutive days				
		- Exclusion criteria: prior history of CL or antimony use, evidence of mucosal or disseminated disease, pregnancy or breastfeeding; HIV or any systemic severe disease						
Soto, 2008 [38]	Open label, RCT in Palos Blancos, Bolivia	- Males and females	≥12	Intramuscular	*L. braziliensis*	Cure rate at 1, 3, and 6 mo; adverse events	44	18
		- Inclusion criteria: a skin ulcer confirmed to be caused by *Leishmania* by visualization of parasites in lesion material by Giemsa staining; either sex; ≥12 yr of age		Pentavalent antimony (Glucantime, 20 mg/kg/d) for 20 d				
		- Exclusion criteria: mucosal disease or anti-leishmanial therapy for at least 6 mo; significant concomitant disease by history, physical examination, or blood tests; pregnancy or lactation		Oral miltefosine 2.5 mg/kg/d for 28 d				
Chrusciak-Talhari, 2011 [35]	Phase II/III open label, randomized trial at a dermatology outpatient clinic in Brazil	- Males and females	2-12 and 13-65	Glucantime intravenously at a dose of 20 mg Sb +5/kg/d (age group 13-65 yr) and 15 mg Sb +5/kg/d (age group 2-12 yr) for 20 consecutive days (maximum daily dose of 3 ampoules)	*L. guyanensis, L. braziliensis *(n=3), *L. lainsoni* (n=1)	Cure rate at 1, 2, 4 ,6 mo; adverse events	56	28
		- Inclusion criteria: patients having clinical diagnosis of CL; illness duration of less than 3 mo; visualization of *Leishmania* amastigotes on Giemsa; no previous *Leishmania* treatment		Miltefosine orally at the total target daily dosage of 2.5 mg/kg of BW (maximum daily dose of 150 mg) for 28 consecutive days				
		- Exclusion criteria: evidence of immunodeficiency or antibodies to HIV, pregnancy or patients not willing or unable to use contraceptives during and 3 mo after the end of therapy, ALT, AST ≥ 3× normal reference values, billirubin ≥ 2× reference values, and creatinine and blood urea nitrogen ≥ 1.5× normal reference values, and any evidence of serious underlying disease (cardiac, renal, hepatic, or pulmonary) including serious infection other than CL						
Asilian, 2014 [39]	RCT carried out in Isfahan, Iran	- Inclusion criteria: aged 15 yr or older, presence of less than 4 cutaneous lesions, duration of the lesions less than 1 mo, lesion size less than 3 cm, no previous use of anti-leishmanial drugs	≥15	Glucantime: topical ointment twice in week (maximum 4 wk)	*L. tropica/major*	Cure rate at 1 mo	32	32
		- Exclusion criteria: pregnancy or lactation, evidence of immunodeficiency, medical history of cardiac, renal, or hepatic disease		Miltefosine: once in day for 28 consecutive days				
Soto, 2007 [34]	Trial in Palos Blancos, Bolivia	Not reported	Not reported	Intramuscular MA	*L. braziliensis*	Cure rate at 2, 4, 6 mo; adverse events	45	26
				Miltefosine orally for 28 d				
Khatami, 2012 [37]	Open label RCT carried out in Bam and Mashhad in Iran	Parasitologically proven cases of CL; healthy subjects on the basis of medical history, physical examination and results of blood tests; age 12-50 yr; BW >40 kg	12-50	Intramuscular injections of MA 60 mg/kg daily for 2 wk oral miltefosine 2.5 mg/kg daily for 4 wk	Anthroponotic CL	Cure rate at 1 mo; adverse events	63	75

RCT, randomized clinical trial; BW, body weight; CL, cutaneous leishmaniasis; PCR, polymerase chain reaction; HIV, human immunodeficiency virus; AST, aspartate aminotransferase; ALT, alanine aminotransferase; MA, meglumine antimoniate; *L, Leishmania*.

**Table 2.** Risk of bias assessment

First author and year of publication [Ref]	Random sequence generation	Allocation concealment	Performance bias	Detection bias	Attrition bias	Reporting bias	Other bias	Total
Asilian, 2014 [39]	Unclear: the scheme of randomization not reported	Unclear: not reported	Unclear	Unclear	Low risk	Low risk	Unclear	Unclear
Rubiano, 2012 [36]	Low risk: computerized balanced block randomization	Low risk: treatment was assigned by the coordinating center via phone call from the study site at subject inclusion	High risk: open label (because of the different routes of administration of the study medications and the unjustified and unethical risk of injection placebo)	Low risk: masked evaluation of outcome	Low risk: follow-up evaluation at 26 wk were completed by 95.6% of randomized patients (111/116)	Low risk: prospective registration: NCT00487253; results on all outcome measures that were pre specified as relevant was presented	Low risk	Low risk
Khatami, 2012 [37]	Unclear: the scheme of randomization not reported	Unclear: not reported	High risk: open label	Unclear: not reported	High risk: cases out of 75 in the MA arm and 31 cases out of 63 in the miltefosine arm finished the study	Unclear: not reported	Unclear	High risk
Chrusciak-Talhari, 2011 [35]	Low risk	Unclear	High risk: open label	Unclear	Low risk	Low risk: retrospective registration: NCT00600548; results on all outcome measures that were pre specified as relevant was presented	Low risk	Unclear
Vélez, 2010 [12]	Low risk: a list of treatments, generated randomly in blocks of eight (EpiInfo)	Low risk: only the study coordinator had access to the list	High risk: open label	Unclear	Low risk	Low risk: results on all outcome measures that were pre specified as relevant was presented	Low risk	Unclear
Machado, 2010 [28]	Low risk: randomization list obtained with using a computer program	Unclear	High risk: open label	Low risk: masked evaluation of outcome	Low risk	Low risk: retrospective registration: NCT00600548; results on all outcome measures that were pre specified as relevant was presented	Low risk	Unclear
Soto, 2008 [38]	Unclear: not reported	Unclear: not reported	High risk: open label	Unclear	Unclear	Unclear: prospective registration: NCT00233545; not all pre specified outcomes were reported	Low risk	Unclear
Soto, 2007 [34]	Unclear	Unclear	Unclear	Unclear	Unclear	Unclear	Unclear	Unclear
Rahman, 2007 [11]	High risk: non-randomized	High risk	High risk: open label	Unclear	Low risk	Low risk: results on all outcome measures that were pre specified as relevant was presented	Unclear	High risk
Mohebali, 2007 [33]	Low risk: balanced block randomization	Unclear	High risk: open label	Unclear	Low risk	Low risk: results on all outcome measures that were pre specified as relevant was presented	Low risk	Unclear

MA, meglumine antimonite; NCT, national clinical trial.

**Table 3.** Results of meta-analyses (comparison of the efficacy of miltefisine and meglumine antimoniate)

Follow up period after end of treatment (d) [Ref]	ITT (RR, RD) [95% CI] (heterogeneity, %)	Effect measure (RR, RD) [95% CI] (heterogeneity, %) after excluding
End of treatment [11,28,35,36,38,39^2^]	RR: 1.25 (0.83, 1.78); n=448; I^2^=64	RR: 1.19 (0.74, 1.93); n =384; I^2^=67
	RD: 0.08 (-0.04, 0.20); n=448; I^2^=71	RD: 0.06 (-0.06, 0.20); n=384; I^2^=72
14 [33]	RR: 1.10 (0.80, 1.51); n=63	
	RD: 0.07 (-0.15, 0.29); n=63	
30 [35,37-39^2^]	RR: 1.23 (0.76, 1.98); n=348; I^2^=86	RR: 0.98 (0.69, 1.39); n=284; I^2^=68
	RD: 0.07 (-0.14, 0.30); n=348; I^2^=84	RD: -0.06 (0.19, 0.14); n=284; I^2^=65
60 [28,34,35]	RR: 1.26 (1.05, 1.50); n=245; I^2^=84	
	RD: 0.16 (0.04, 0.28); n=245; I^2^=0	
90 [11,12^2^,^33^,^36^,^38^]	RR: 1.03 (0.85, 1.24); n=559; I^2^=75	RR: 1.12 (1.00, 1.27); n=271; I^2^=11
	RD: 0.03 (-0.12, 0.18); n=559; I^2^=77	RD: 0.10 (0.01, 0.20); n=271; I^2^=14
120 [34,35]	RR: 1.07 (0.84, 1.36); n=155; I^2^=39	
	RD: 0.05 (-0.08, 0.19); n=155; I^2^=18	
180^1^ [11,12^2^,^28^,^33^-^36^,^38^]	RR: 1.01 (0.92, 1.11); n=804; I^2^=39	RR: 1.14 (1.03, 1.27); n=516; I^2^=35
	RD: 0.01 (-0.05, 0.07); n=804; I^2^=60	RD: 0. 01 (-0.05, 0.07); n =516; I^2^=60
180 [11,12^2^,^28^,^33^-^36^,^38^]	RR: 1.05 (0.91, 1.20); n=804; I^2^=56	RR: 1.10 (0.97, 1.25); n=516; I^2^=35
	RD: 0. 04 (-0.05, 0.15); n=804; I^2^=60	RD: 0.08 (-0.01, 0.16); n=516; I^2^=26

ITT, intention to treat; RR, relative risk; RD, risk difference; CI, confidence interval.

1 Fixed method.

2 Excluded studies.

**Table 4.** Comparison of the efficacy of miltefisine and meglumine antimoniate regarding to parasite species

Parasite species [Ref]	ITT (RR, RD) [95% CI] (heterogeneity, %)
*L. tropica/major, L. panamensis/major, L. guyanensis, L. braziliensis* [11,12,28,33-36,38]^1^	RR: 1.14 (1.03, 1.27); n=804; I^2^=35
	RD: 0.01 (-0.05, 0.07); n=804; I^2^=60
*L. tropica/major, L. panamensis/major, L. guyanensis, L. braziliensis* [11,12,28,33-36,38]	RR: 1.05 (0.91, 1.20); n=804; I^2^=56
	RD: 0.04 (-0.05, 0.15); n=804; I^2^=60
*L. tropica/major* [11]	RR: 1.30 (0.86, 1.95); n=30
	RD: 0.20 (-0.09, 0.49); n=30
*L. panamensis* [36]	RR: 1.20 (0.97, 1.47); n=116
	RD: 0.13 (-0.01, 0.29); n =166
*L. major* [33]	RR: 1.01 (0.79, 1.28); n=63
	RD: -0.01 (-0.18, 0.20); n=63
*L. guyanensis* [35]	RR: 1.33 (0.90, 1.95); n=84
	RD: 0.17 (-0.04, 0.39); n=84
*L. braziliensis* [12,28,34,38]	RR: 0.92 (0.82, 1.04); n=511; I^2^=0
	RD: -0.05 (-0.13, 0.02); n=511; I^2^=0
*L. tropica/major, L. panamensis/major, L. guyanensis* [11,33,35,36]^1^	RR: 1.18 (1.02, 1.37); n=293; I^2^=0
	RD: 0.12 (0.02, 0.22); n=293; I^2^=0
*L. tropica/major, L. panamensis/major, L. guyanensis* [11,33,35,36]	RR: 1.15 (1.01, 1.32); n=293; I^2^=0
	RD: 0.11 (0.01, 0. 21); n=293; I^2^=0

ITT, intention to treat; RR, relative risk; RD, risk difference; CI, confidence interval; *L, Leishmania*.

1 Fixed method.

**Table 5.** The results of meta-regression

Factors	Level	Coefficient	t-value	p-value	I-squared (%)	tau2	Adjusted Rsquared (%)
Country category		0.04	0.25	0.81	10	0.03	-25.13
Sex ratio		0.29	0.56	0.59	14	0.03	-25.15
Sample size		0.00	-1.50	0.18	6	0.02	25.41
Injection type		0.03	2.19	0.07	13	0.01	53.98
Quality of study	Low	Reference	-	-	3	0.02	1.16
	Unclear	-0.17	-0.77	0.47			
	High	0.10	0.29	0.78			
