# Supplementary material for: Efficacy of miltefosine compared with glucantime for the treatment of cutaneous leishmaniasis: a systematic review and meta-analysis
Source: Epidemiol Health. 2019 Mar 31;41:e2019011. doi: 10.4178/epih.e2019011 (PMC6635659; doi:10.4178/epih.e2019011)
Supplement: Supplementary file 1 [file epih-41-e2019011-supplementary.pdf]

Supplementary Material 1. The PubMed systematic search strategy

((((((“miltefosine" [Supplementary Concept] OR "miltefosine transporter, Leishmania donovani" [Supplementary Concept])) OR (((n-hexadecylphosphorylcholine OR HDPC OR hexadecylphosphocholine OR Milte* OR Impavido OR “D 18506 ”OR D18506 OR D-18506 OR Hexadecylphosphorylcholine OR (Phosphorylcholine AND hexadecyl)))))) AND ((("Meglumine antimonite" OR "N-methylglucamine antimonite" OR Glucanti* OR "antimony n methylglucamine" OR" methylglucamine antimon*" OR "n methylglucamine antimon*" OR "n methylglucamine antimonic acid" OR protosib OR "rp 2168" OR "wr214975 aj" OR "pentavalent antimonials")) OR "meglumine antimoniate] "Supplementary Concept]))) AND ((("Leishmania mexicana"[Mesh] OR" Leishmania braziliensis"[Mesh] OR "Leishmania major"[Mesh] OR "Leishmania tropica"[Mesh])) OR ("skin leishmaniasis" OR "Cutaneous Leishmanias*" OR (leishmaniases AND cutaneous) OR "Oriental Sore" OR (sore AND oriental) OR (leishmaniasis AND "Old World") OR "Old World Leishmaniasis" OR (leishmaniasis AND "New World") OR "New World Leishmaniasis" OR (leishmaniasis AND american) OR "American Leishmaniasis" OR ("Cutaneous Leishmanias*" AND diffuse) OR "Diffuse Cutaneous Leishmanias*" OR (leishmaniases AND "Diffuse Cutaneous") OR" Aden ulcer" OR "Aleppo boil" OR "Bagdad sore" OR "Borovskii disease" OR" cafaneous leishmaniasis" OR "chiclero ulcer" OR "chiclero ulcus" OR" cutaneous leishmaniasis" OR "cutaneous leishmaniosis" OR "Delhi boil "OR "dermal leishmaniasis" OR "epidermic leishmaniasis" OR "leishmaniasis cutanea" OR "leishmaniasis cutis" OR "leishmaniasis nodosa" OR (leishmaniasis AND cutaneous) OR "diffuse cutaneous" OR leishmaniasis OR (leishmaniasis AND skin) OR "Penjdeh ulcer" OR "Penjdeh ulcus" OR" Syrian ulcer" OR "Turkestan ulcer" OR "Turkestan ulcus" OR (ulcer AND chiolero))) AND 1991/01/01:2017/07/31[DP]

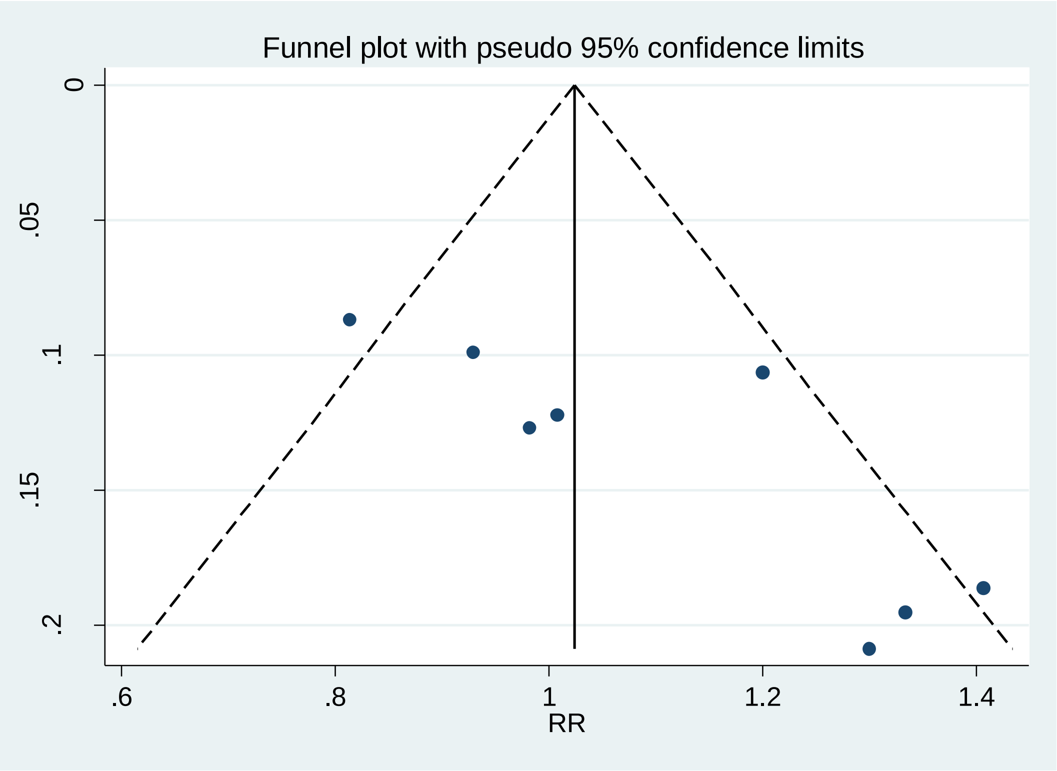

Supplementary Material 2. Publication bias assessed by Funnel plot.

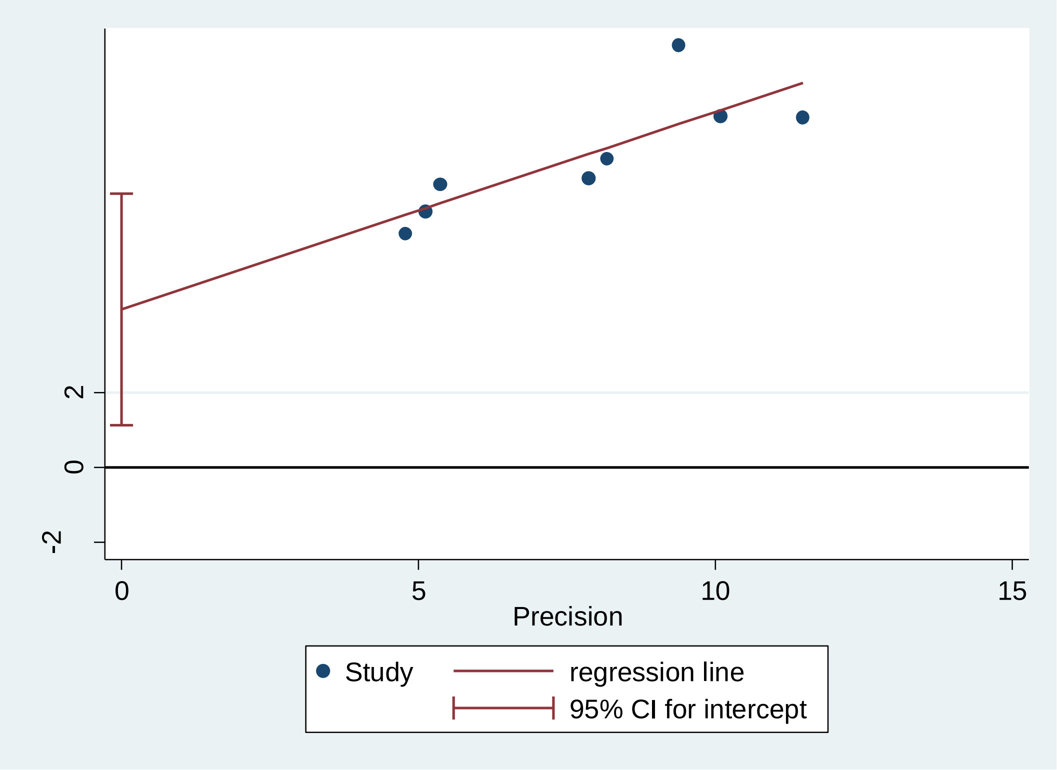

Supplementary Material 3. Publication bias assessed by Egger's method.

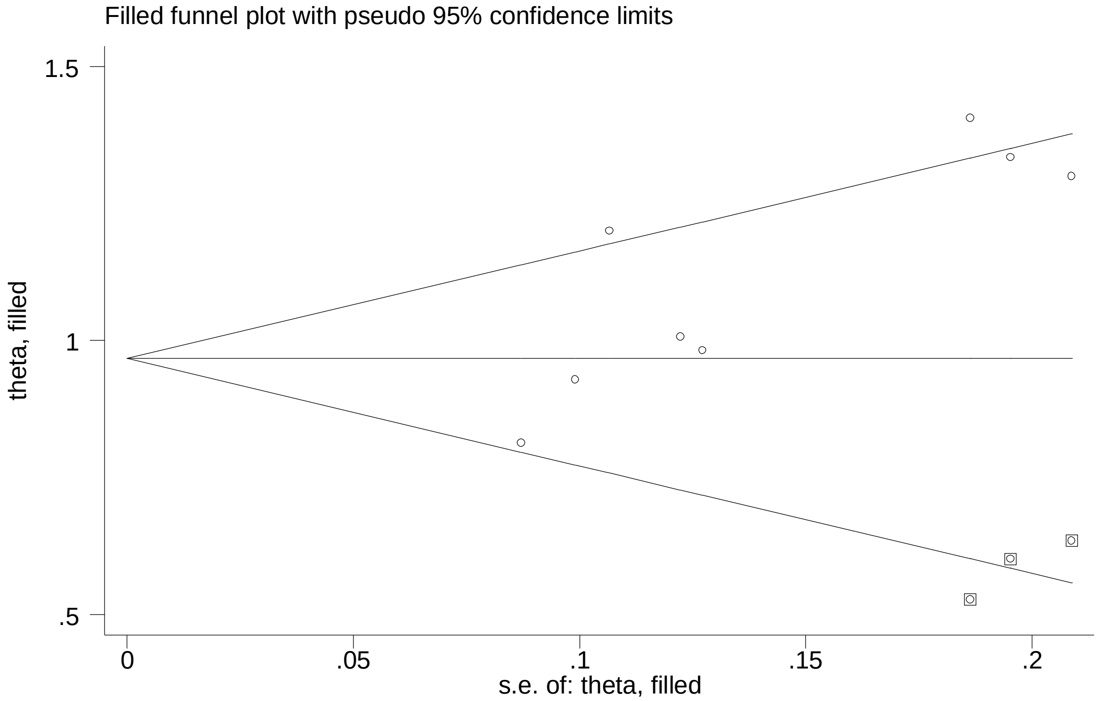

Supplementary Material 4. Publication bias assessed by trim and fill method. Hollow cycle in box represents the estimated missing study.
